# Soft robotics in wearable and implantable medical applications: Translational challenges and future outlooks

**DOI:** 10.3389/frobt.2023.1075634

**Published:** 2023-02-08

**Authors:** Linda Paternò, Lucrezia Lorenzon

**Affiliations:** ^1^ The BioRobotics Institute, Scuola Superiore Sant’Anna, Pisa, Italy; ^2^ Department of Excellence in Robotics and AI, Scuola Superiore Sant’Anna, Pisa, Italy

**Keywords:** soft robotics, limb prostheses, soft exoskeleton, implantable device, artificial organ, biorobotics, wearable-user interaction, physical human machine interaction

## Abstract

This work explores the recent research conducted towards the development of novel classes of devices in wearable and implantable medical applications allowed by the introduction of the soft robotics approach. In the medical field, the need for materials with mechanical properties similar to biological tissues is one of the first considerations that arises to improve comfort and safety in the physical interaction with the human body. Thus, soft robotic devices are expected to be able of accomplishing tasks no traditional rigid systems can do. In this paper, we describe future perspectives and possible routes to address scientific and clinical issues still hampering the accomplishment of ideal solutions in clinical practice.

## Introduction

In the past, rigid robotic systems have been the main research focus in traditional robotics due to their ability to provide high output forces and perform fast and precise position control tasks. However, a world in which robotic systems should directly interact with humans must drastically switch its focus towards new human-centered requirements to guarantee a safe physical human-robot interaction (Trimmer 2014; Murphy 2022).

In robotic systems, safety can be achieved through specific planning and control strategies, such as working on robot navigation, collision detection and avoidance, stiffness and force control. Otherwise, it can rely on specific mechanical design rules (Pervez and Ryu 2008). Focusing on this second approach, soft robotics opens up novel possibilities for bridging the gap between humans and machines (Kim et al., 2013; Wood and Walsh 2013; Laschi et al., 2016). Indeed, soft robotics aims to develop “systems that are capable of autonomous behavior, and that are primarily composed of materials with moduli in the range of that of soft biological materials” (Rus and Tolley 2015). Thus, the soft paradigm turns out especially intriguing for biorobotic applications that require close interactions with the human body (Cianchetti et al., 2018).

Against this background, this work analyses the current challenges and future perspectives of soft robots in wearable and implantable medical applications. Starting from the state-of-the-art in these fields, novel possible scenarios are figured out based on the exploitation of the advantages of the soft robotic approach and with the awareness that “soft robots will be capable of performing feats no current machines can accomplish” (Trimmer 2013).

## Physical interfaces in wearable robotics

In the last decades, the development of ever more sophisticated wearable robots, such as limb prostheses and exoskeletons, has made it possible to transcend several disabilities through technological innovation. To accomplish this task and even more, achieving a physical and cognitive symbiosis of the device with the wearer is the key aspect (Pons 2019).

Focusing on the physical interaction of wearable robots, huge research efforts are still required in the design area since “we still do not understand how to attach devices to the body, mechanically” (Herr, 2014). An ideal physical Human-Machine Interface (pHMI) should enable an unobtrusive biomechanical coupling with the user’s body, resulting similar to a “second skin” (Herr 2009). It should allow for proper alignment with natural joints and safe force transmissions, avoiding kinematic incompatibilities and motion restrictions. Furthermore, it should be comfortable. Indeed, even the most brilliant and advanced system is useless if comfort is not guaranteed.

Current wearable pHMIs are rigid and passive structures. Although a soft layer is usually interposed between rigid materials and natural tissues, the unusual normal and shear stresses applied on the skin often lead to lesions and pain, and alter blood circulation. Volume and shape changes of the body—caused by fluid movements or muscle contraction/relaxation—affect the interface fitting, thus causing relative movements and altering in turn the stress distribution on tissues. The artificial physical barrier between the skin and the environment prevents the physiological thermal transfer mechanisms, thus affecting the homeostasis of the body and increasing the sweating rate. These aspects are the cause of several discomforts, dermatological and vascular complications, and, in the worst cases, device abandonment (Herr 2009; Wood and Walsh 2013).

Solving these issues is particularly urgent in the prosthetic field, especially in lower limb amputees due to the higher loads involved at the residual limb/prosthesis interface, namely the socket (Paternò et al., 2018). Osseointegration is a recent method that allows for connecting the prosthesis directly to the patient’s bones, thus restoring the load transmission through the skeleton and overcoming socket-related issues. Even though it is the most promising approach, it is invasive and not easily acceptable by patients. Furthermore, several challenges have still to be faced, especially in terms of infection risks due to its percutaneous nature (Overmann and Forsberg 2020). Thus, smart prosthetic sockets integrating sensing and actuation technologies able to optimize fitting and stress distribution on tissues in an automatic and closed-loop fashion could be an alternative successful solution (Sanders et al., 2019; Weathersby et al., 2021).

**FIGURE 1:**
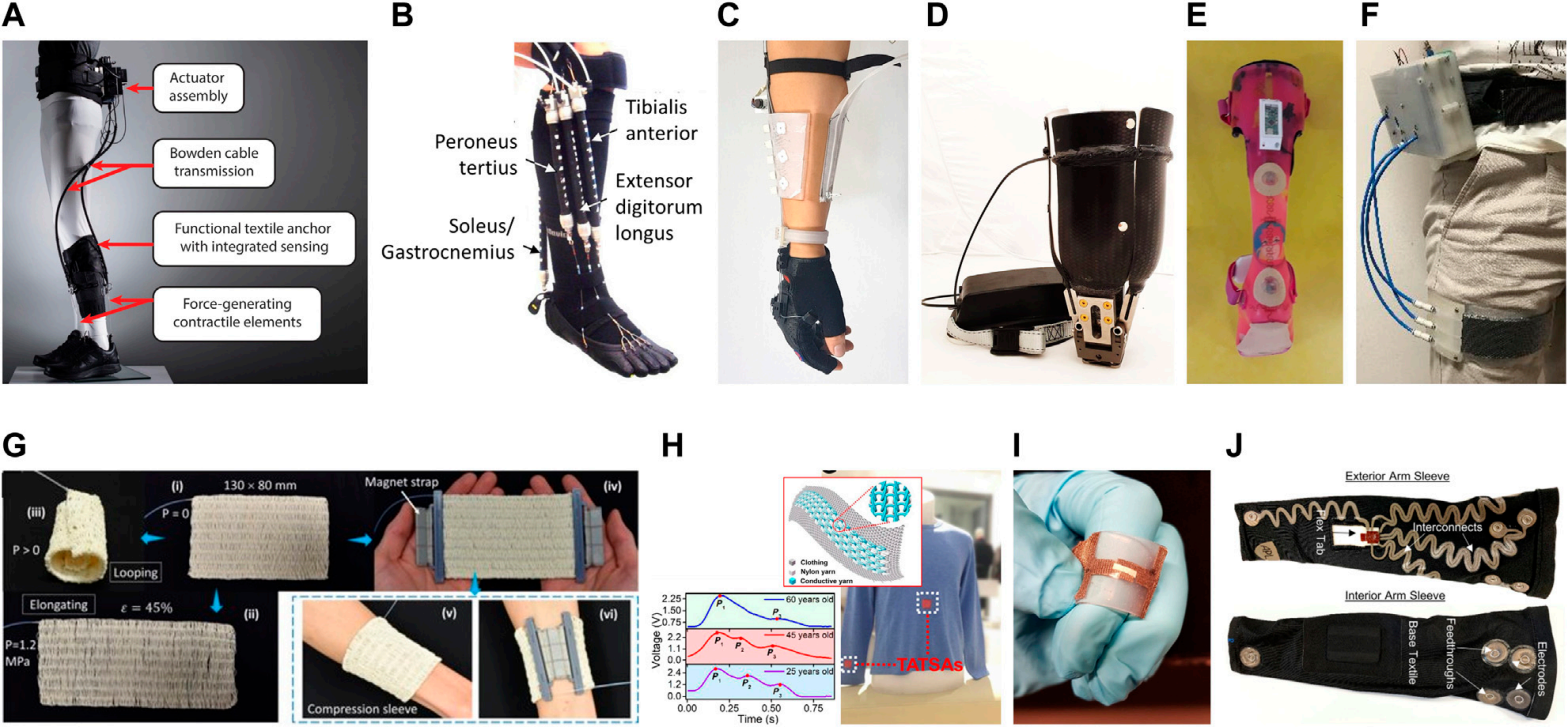
**(A)** Soft robotic exosuit for assistive and rehabilitation applications in post-stroke patients (Awad et al., 2020) (republished from Koch and Font-Llagunes, 2021); **(B)** Wearable device for ankle–foot rehabilitation based on McKibben pneumatic artificial muscles (Park et al., 2014) (republished from Koch and Font-Llagunes, 2021); **(C)** Soft wrist-wearable robot with a motor-driven tensioning system to improve force transmission (Choi W. H. et al., 2019) (courtesy of Professor Kyu-Jin Cho, Seoul National University, South Korea); **(D)** Motor-driven adjustable prosthetic socket for transfemoral amputees (Paterno et al., 2021) (courtesy of Professor Arianna Menciassi, Scuola Superiore Sant’Anna, Italy); **(E)** Sensorized orthosis made by vacuum lamination of a stretchable sensor system (Bauwens et al., 2020) (courtesy of Professor Jan Vanfleteren, Ghent University, Belgium); **(F)** Variable stiffness and shape orthosis (Ibrahimi et al., 2021) (courtesy of Professor Arianna Menciassi, Scuola Superiore Sant’Anna, Italy); **(G)** Active textiles based on fluid-driven artificial muscle fibers (republished from Phan et al., 2022); **(H)** Triboelectric All-Textile Sensor Array (TATSA) for epidermal pressure measuring (Fan et al., 2020) (republished from Tseghai et al., 2020); **(I)** Stretchable and washable strain sensor for human motion monitoring (republished from Tolvanen et al., 2018); **(J)** E-textile suit for surface electromyography for level-of-effort estimation (republished from Ohiri et al., 2022). All figures are republished under the Creative Commons Attribution 4.0 International License (http://creativecommons.org/licenses/by/4.0/).

Recently, in the exoskeletons field, great steps forward have been carried out thanks to the exploitation of the soft robotics paradigm. Different solutions based on textile anchors (Figure 1A) and soft actuators (Figure 1B) have been developed, enabling designs more similar to suits than to robots (Wood and Walsh 2013; Masia et al., 2018; Walsh 2018). These designs show great advantages, especially in terms of physical interaction, avoiding the need for precise alignments with the user’s natural joints and eliminating movement restrictions due to weight and encumbrance. However, rigid systems remain the only available solution in some specific rehabilitation domains, and there is still great room for improvement to achieve real biomimetic systems able to meet the users’ acceptance, particularly in terms of comfort (Pons 2019; Xiloyannis et al., 2021).

In this context, the blending of soft robotics and material science has a special potential to enable wearable interfaces with new capabilities mainly based on the tunability of the mechanical properties of their constitutive materials and the morphological adaptability of their structures. The constant progress in CAD-CAM techniques offers an attractive pathway toward the development of bioinspired materials with even more complex architectures (Studart 2016). Heterogeneous multifunctional composite materials could be used at the pHMIs to obtain stiffness gradients and damping properties. These interfaces could be designed and developed according to the properties of the underlying tissues and limb shape (Sengeh et al., 2013; Petron et al., 2017; Paternò et al., 2020). They could be combined with actuation systems to compensate for major volume and shape changes, thus guaranteeing proper fitting, over time. Along this path, the active pHMI of the wrist-wearable robot proposed by Choi H. et al. (2019) offers a promising solution that combines a multi-layer soft structure with a motor-driven corset for efficient fitting and force transmission (Figure 1C). Similarly, motor-driven sockets combined with silicone socks, namely liners, have been proposed in the prosthetic domain (Figure 1D).

Alternatively, active interfaces able to modulate their stiffness and damping properties in a wide range could be integrated to improve the interaction with the biological tissues (Kornbluh et al., 2004). They could allow for a stiffened interface of the device when biomechanical stability is really needed, otherwise for a soft, compliant, and comfortable interaction. Technologies with a pseudo-planar design, such as layer jamming based on vacuum (Choi H. et al., 2019; Paterno et al., 2022) or electrostatic forces, are highly promising in this sense (Kornbluh et al., 2004; Herr 2014; Wang et al., 2019). In addition, the integration of vacuum jamming technology with soft pneumatic actuators has already demonstrated the potential to enable tunable stiffness and shape-changing interfaces able to improve both comfort and fitting of wearable devices (Figure 1F) (Ibrahimi et al., 2021). Pneumatic systems can benefit from many energy sources compatible with wearable application requirements (Wehner et al., 2014). Moreover, the development of textile-based pneumatic energy harvesting system (Shveda et al., 2022) or the use of soft valves that can control airflow by acting as switches for automated functions (Rothemund et al., 2018; Kendre et al., 2022) have paved the way for overcoming the main challenges of current bulky and expensive commercial mechatronic components for pneumatic systems. In this scenario, active fluidic textiles based on pneumatic artificial muscle fibers can represent a successful soft actuation solution (Figure 1G) (Kurumaya et al., 2019; Zhu et al., 2019; Phan et al., 2022). Indeed, they could be combined in pseudo planar design with a great variety of smart fabric sensors and e-textile technologies (Figures 1H–J) to achieve a pHMI able to adapt to the physiological changes of the body, over time (Castano and Flatau 2014; Islam et al., 2020).

The pHMI of wearable robots must be equipped with sensors and actuators also to allow for intention decoding and sensory feedback functionalities. Indeed, a closed human-centered control loop involving efferent motor outputs and afferent sensory inputs is fundamental to integrate the device with the user, synergically. Focusing on non-implantable bidirectional physical interfaces, some technologies can be integrated into the external rigid parts of wearable robots since direct contact with the user’s tissues is not required (e.g., sensor network based on IMUs) (Lotti et al., 2022). Since rigid parts usually replicate the user’s body shape and are made of thermoformed materials, stretchable electronics can be employed to obtain compact and light designs, as shown in Figure 1E (Bauwens et al., 2020). Otherwise, solutions based on soft and compliant materials, such as textiles or elastomers, have to be used if contact with tissues is needed (e.g., EMG sensors) (Figures 1H–J).

The integration of several technologies in physical wearable interfaces can be stated as the main challenge. In this regard, the recent advances in manufacturing processes, material and computer science, miniaturized and stretchable electronics have been pushing forward the evolution of a novel class of materials that can integrate computing and communication components, sensors, and actuators in increasingly compact and tight designs (McEvoy and Correll 2015). These so-called “robotic materials” can change not only their stiffness but also their shape or other physical parameters, and they can do this in a fully programmable way. The robotic material concept is based on the exploitation of miniaturized technologies that can sense material properties, process them, and activate desired actions. All these technologies have to be integrated into a continuous structure created by the material itself. This approach is in its infancy but the great progress in textile engineering, soft robotics, and smart material science in the last decades has opened up several novel avenues to accomplish this goal (Sanchez et al., 2021). By using soft and comfortable materials as continuous structures in contact with the user’s body, the route of robotic materials is particularly intriguing for the development of wearable interfaces that are not just passive and rigid structures, but robotic skins able to sense, exchange information with the user, and self-adapt in real-time and in a closed-loop fashion to the full spectrum of the wearer’s needs.

## Bio-integration of implantable robotic devices

**FIGURE 2:**
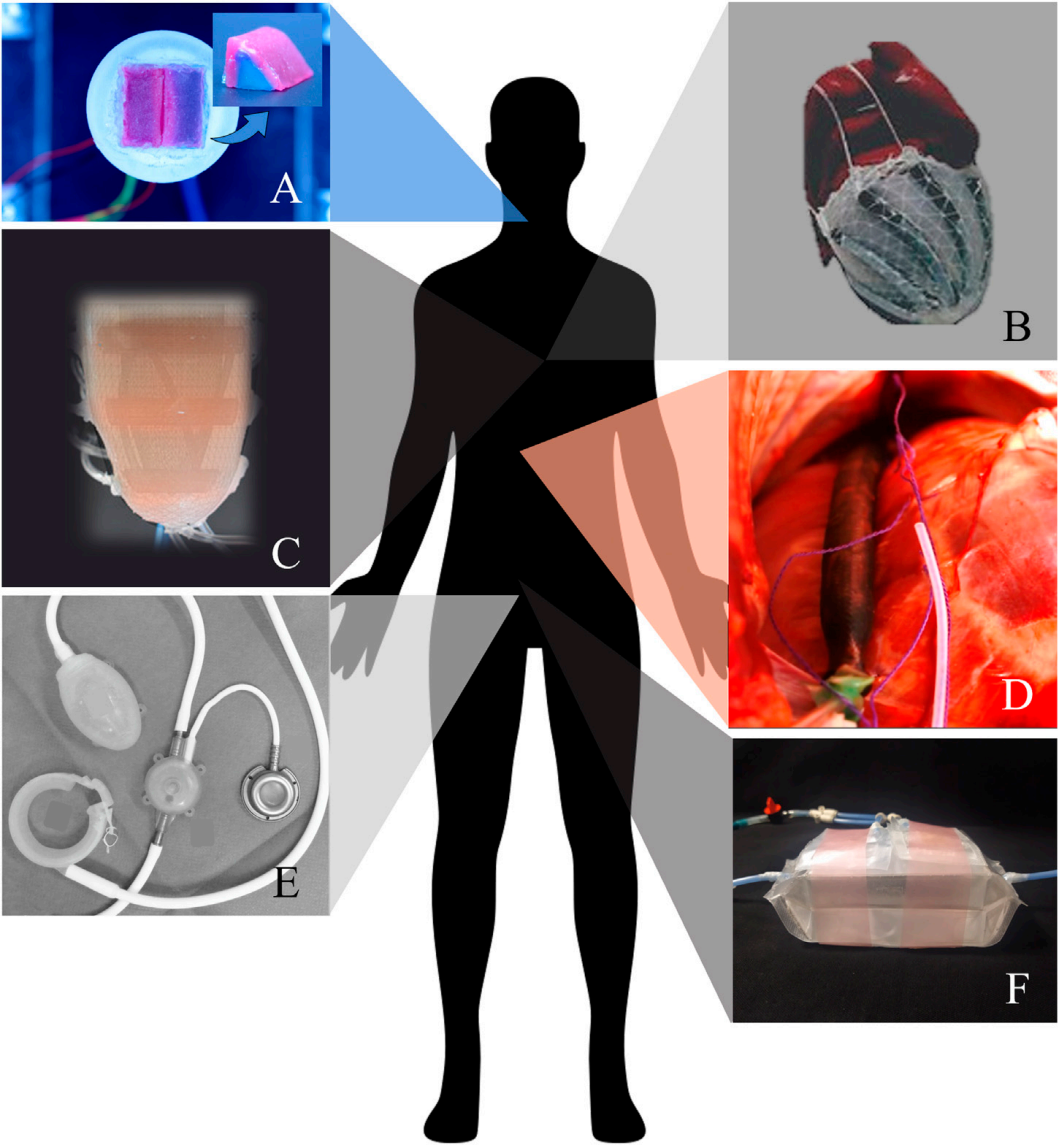
**(A)** Synthetic vocal folds simulator (courtesy of Dr Martina Maselli, Scuola Superiore Sant’Anna, Italy); **(B)** Soft robotic sleeve, pneumatically actuated by McKibben artificial muscles (republished from Park et al. (2022)); **(C)** Soft robotic left ventricle prototype, pneumatically actuated by a dual-layer of flattened and cylindrical McKibben actuators (republished from Park et al. (2022)); **(D)** Soft robotic artificial ventilator: a close-up on a supradiaphragmatic McKibben actuator placed on the right hemidiaphragm of an *in vivo* porcine model (republished from Hu et al., 2021); **(E)** Soft Anal Band System (Agency for Medical Innovation, Austria), hydraulically actuated (republished from Goos et al., 2013). **(F)** Soft robotic artificial bladder, hydraulically actuated by bellows actuators (Casagrande et al., 2022) (courtesy of Ms Giada Casagrande, Scuola Superiore Sant’Anna, Italy). All figures are republished under the Creative Commons Attribution 4.0 International License (http://creativecommons.org/licenses/by/4.0/).

Less than 10 years ago, the biomedical field experienced an unprecedented development of high-fidelity physical organ simulators with various applications, such as the training of aspiring practitioners and surgeons, and the extensive testing of novel biomedical devices and medical instrumentations (Maglio et al., 2021). Undoubtedly, this recent growth of human body-parts simulators has been strongly encouraged and accompanied by the concurrent establishment of soft robotics principles and technologies. Firstly, the engineering of long-lasting materials with tunable mechanical properties, such as elastomers, allowed an accurate replication of not only the haptic perception of the organ but also of its natural dynamics in the body. To give just one example, in 2021, Conte et al. (2021) presented a dynamic and passive vocal folds (VFs) simulator made by three different silicone materials, to mimic the natural multilayer morphology of the VFs (see Figure 2A). The geometry replicates the main features of the natural counterpart and the materials have mechanical properties that approximate the ones of the natural tissues, enabling the simulation of the vibratory characteristics of both healthy and pathological VFs.

When the target body part has a natural motility given by the contraction of muscle fibers, the simulation of the organ physiology requires constitutive materials that have similar mechanical properties, and that are active and stimuli-responsive too. In this scenario, the integration of soft actuating technologies resulted to be game-changing, and the natural motion of several human organs was reproduced (Gong et al., 2021). In the past decade, outstanding results were achieved using soft robotics in cardiovascular therapy (Wamala et al., 2017). A significant branch of studies started from the development of a soft and active material in which an elastomeric matrix embeds McKibben artificial muscles (Roche et al., 2014). By helically disposing the actuators in a 3D shape, the dominant muscle layer of the heart myocardium was represented. As a result, upon actuation, the motion of the structure accurately simulated the apical twist of a human left ventricle. Lately, this bioinspired actuation strategy was implemented in a soft robotic sleeve that conforms around the natural heart to provide cardiocirculatory support (see Figure 2B) (Roche et al., 2017). The adaptability demonstrated in the interaction with the natural heart both *ex vivo* and *in vivo* would not have been possible either with rigid components or with compliant materials only; the introduction of soft robotics technologies enabled the controllable and dynamic change of material properties in relation to physiological needs.

For implanted devices, the surface interaction with biological fluids, especially the blood, is among the biggest challenges that need to be faced. In fact, the human body reacts to intimate contact with a foreign body by activating an inflammatory response, that leads to the formation of fibrotic tissue and/or the coagulation cascade, with consequent thromboembolic complication. For cardiocirculatory devices, if one possibility is to avoid direct contact with blood, the consequent design space limitation probably leaves no option to those patients that, being affected by end-stage heart failure, need a partial, or even a total, organ replacement (Cohrs et al., 2017). In 2022, Roche et al. presented a design framework for optimizing both the biomechanics and the hemodynamics of a soft robotic synthetic ventricle (Figure 2C) whose functionality is within the physiological range of a healthy left ventricle (Park et al., 2022). However, prior to the implementation of the ventricle design for *in vivo* applications, the performances need to be verified in a simulator of human circulation, and a comprehensive assessment of the thrombogenic risk associated with the ventricle implant must be performed.

Additional considerations can be made on the possibility of adjusting the functionality of a soft robotic device in relation to the body’s needs, an aspect of paramount importance that requires the integration of sensor technologies and control units (Polygerinos et al., 2017). Very recently, exciting results on the synchronization with the native respiratory efforts were shown by an implantable ventilator that consists of two McKibben actuators placed on the diaphragm of the patient and anchored to the ribs (Figure 2D) (Hu et al., 2021).

The intimate functional and biological integration of an implantable soft system with the human body is a fundamental requirement, whose fulfilment cannot forget the design of robot-to-body interfaces and the selection of suitable materials. While the research for the development of a bio-adhesive or a bio-integrating component will be crucial in the near future to guarantee a safe and stable mechanical coupling of a soft robotic implant with the natural tissues, several biocompatible elastomers, whether for short- or long-term implants, are readily available in the market (www.elkem.com). However, their poor availability, the higher costs of the materials, and the expensive equipment needed for manufacturing are the limiting factors that prevent the adoption of biocompatible materials at the early stages of development. Certainly, the use of biocompatible materials is essential but not satisfactory, when the objective is a complete bio-integration of a soft robotic device in the human body. From this perspective, the research on the tissue engineering of elastomeric components is still in its infancy, and it is believed to be one of the most promising paths to follow.

However, the biocompatibility of an implanted soft system is only one aspect of the broad safety requirement. Firstly, an implanted device must be reliable, and, in the case of long-term implants, longevity must be proven too. In this regard, the most demanding organ is undoubtedly the heart, beating over 30 million times per year. A candidate artificial heart is required to be durable at over 100 million beats, at physiological heart rates. In the state-of-the-art, at present, none of the presented soft robotic artificial hearts reached this exigent target of longevity (Guex et al., 2020).

The reliability and the longevity of soft robotics systems are both aspects strictly related to the employed materials and the manufacturing techniques. Currently, fabrication methods such as casting, injection moulding and 3D printing, allow the realization of disparate soft structures. Nevertheless, the problems of precision, scalability, multi-material fabrication, and in some cases very complex inner structures have not found a definite solution yet (Wei and Ghosh 2022). Sometimes, the difficulties encountered in manufacturing, especially where complex geometries and soft-to-rigid interfaces are involved, are really the limiting factor to a clear demonstration of a technology that works perfectly, but on simulation only.

Finally, a well-known bottleneck for active artificial organs and assistive devices that are meant to be fully implanted is represented by powering and energy harvesting (Menciassi and Iacovacci 2020). For what concerns powering, the internal combustion strategy was explored in a heart-inspired soft pump (Schumacher et al., 2014). However, combustion-based pneumatic power supplies are poorly compatible with implantable applications. Indeed, they have the drawbacks of limited safety, high local temperatures, and toxic byproduct generation, which also require extensive system-level development. In the present scenario, the most investigated powering in soft robotics is fluidic (Cianchetti et al., 2018), as can be appreciated from the examples reported in Figure 2. Fluidic soft systems have limited portability, owing to the need for a source of compressed air or fluid. Battery-based diaphragm microcompressors are commercially available and have been used to power fluidic soft systems (Wehner et al., 2014). Diaphragm microcompressors have a relatively high capacity, but they only provide low flow rates and maximum pressures, additionally being noisy and bulky. At present, commercial microcompressors would struggle at operating at high-speed large soft robotic devices, such as artificial hearts. Moreover, a fully implanted device is not accessible for battery replacement. In this case, the gold standard is the integration of a lithium-ion battery, which has high energy and power densities and is available in many form factors and capacities. To overcome battery duration issues, wireless energy transfer systems can be employed to charge the implanted batteries. In the future, further efforts in realizing miniaturized, highly efficient and safe pumps and batteries, together with energy transfer systems, will provide the missing tassels for a realistic translation into clinics of implantable soft robotic devices.

The reported considerations and the state-of-the-art examples undeniably highlight the great potential of soft robotics in the advancement of implantable assistive devices and artificial organs. At the same time, definitive solutions are still needed for several big challenges and multidisciplinary research is recognized as the key to tackling such complex problems. The broad research that has been conducted in the last 10 years on high-fidelity organ simulators is recognized as pioneering, not only for the understanding of human pathophysiology (Figure 2A) but also for the development of artificial devices for organ replacement. Indeed, the fundamental research on building blocks, such as material chemistry and actuating elements, and the realization of a synthetic organ simulator can, at least, bring very close to an artificial organ. Nevertheless, additional multidisciplinary efforts are still needed for implanting soft artificial organs that are proven to be effective, safe, and long-lasting, finally making the possibility of clinical translation real. To give a future perspective, soft robotics artificial organs and assistive devices will have a role in the sustaining or substitution of those human body functions that are almost purely mechanical. In Figure 2, we show some examples of the most advanced implantable soft robotic devices that were developed to sustain the blood pumping (b, c), the diaphragmatic movement involved in respiration (d) and the sphincteric (e) and the excretory (f) action.

## Conclusion

Recent research advancements in wearable and implantable medical applications allow for replacing or restoring several functionalities of the human body, ever more effectively. However, several issues have still to be faced to overcome current translational challenges. Focusing on the physical human-robot interaction, wearable systems have to ensure both stable and comfortable biomechanical coupling with the user’s body for guaranteeing safety and usability. Even if wearable robotics has enabled a huge breakthrough in the treatment of patients with motor impairments, the design of an effective physical interface still represents an open issue in this field. Regarding implantable devices they have to be effectively integrated within the human body, from a functional, morphological and biological point of view. Nevertheless, huge efforts are still needed in the search for responsive, safe, and durable soft materials that will constitute the building blocks of the next-generation of soft artificial organs.

“How do you make a robot that is strong enough to be useful and safe to use? How can you make a robot that works in an unpredictable or changing environment? How can you communicate with a robot in a natural and effective way?” (Trimmer 2014). In this scenario, it can be certainly state that soft robotics provides unique features for paving the way for novel classes of robotic systems able to accomplish these needs. Thus, the soft paradigm will certainly play a key role in future wearable and implantable robotic applications to enable solutions that can interact and adapt to the anatomy and to the physiology of the patient, in a dynamic and safe manner.

Nowadays, the development of a robot that interacts intimately with the human body is one of the biggest challenges of science. The ever changing, and in some cases hostile, application environment represented by the human body defines a large number of demanding requirements that span among effectiveness, bio-integration, safety, and durability. In this context, calls for innovative technological solutions are open in several science fields, such as engineering, material and biological science. Soft robotics has paved the way for a lot of smart and fulfilling solutions that can really have a significant impact on translational medicine in the near future. Thanks to its recent emergence, soft robotics results in an extremely fruitful and attractive field, especially for young researchers. Indeed, the complexity of a soft robot working in close contact with humans needs innovation, enthusiasm, and several years to follow the long path towards the clinical approval of a biomedical device.

## Data Availability

The original contributions presented in the study are included in the article/supplementary material, further inquiries can be directed to the corresponding author.
