# Corrigendum: Soft robotics in wearable and implantable medical applications: translational challenges and future outlooks

**DOI:** 10.3389/frobt.2023.1243121

**Published:** 2023-06-28

**Authors:** Linda Paternò, Lucrezia Lorenzon

**Affiliations:** ^1^ The BioRobotics Institute, Scuola Superiore Sant’Anna, Pisa, Italy; ^2^ Department of Excellence in Robotics and AI, Scuola Superiore Sant’Anna, Pisa, Italy

**Keywords:** soft robotics, limb prostheses, soft exoskeleton, implantable device, artificial organ, biorobotics, wearable-user interaction, physical human machine interaction

In the published article, there was an error in the **Funding** statement.

“A funding agency was missing. The original text is: This work was promoted by INAIL, the Italian National Institute for Insurance against Work-related Injuries (non-commercial entity), within the PR19-PAI-P2-MOTU++ (Protesi robotica di arto inferiore con smart socket ed interfaccia bidirezionale per amputati di arto inferiore: personalizzazione mediante human-in-the-loop optimization) project framework (www.santannapisa.it/en/institute/biorobotics/motu)”.

The correct **Funding** statement appears below.

